# Anticancer Effects of Geopropolis Produced by Stingless Bees on Canine Osteosarcoma Cells *In Vitro*

**DOI:** 10.1155/2013/737386

**Published:** 2013-04-18

**Authors:** Naiara Costa Cinegaglia, Paulo Ricardo Oliveira Bersano, Maria José Abigail Mendes Araújo, Michelle Cristiane Búfalo, José Maurício Sforcin

**Affiliations:** ^1^Surgery and Orthopedics Department, Medical School, São Paulo State University (UNESP), 18618-970 Botucatu, SP, Brazil; ^2^Investigative and Comparative Pathology Laboratory, College of Veterinary Medicine and Animal Husbandry, São Paulo State University (UNESP), 18618-970 Botucatu, SP, Brazil; ^3^Department of Microbiology and Immunology, Biosciences Institute, São Paulo State University (UNESP), 18618-970 Botucatu, SP, Brazil

## Abstract

Geopropolis is produced by indigenous stingless bees from the resinous material of plants, adding soil or clay. Its biological properties have not been investigated, such as propolis, and herein its cytotoxic action on canine osteosarcoma (OSA) cells was evaluated. OSA is a primary bone neoplasm diagnosed in dogs being an excellent model *in vivo* to study human OSA. spOS-2 primary cultures were isolated from the tumor of a dog with osteosarcoma and incubated with geopropolis, 70% ethanol (geopropolis solvent), and carboplatin after 6, 24, 48, and 72 hours. Cell viability was analyzed by the crystal violet method. Geopropolis was efficient against canine OSA cells in a dose- and time-dependent way, leading to a distinct morphology compared to control. Geopropolis cytotoxic action was exclusively due to its constituents since 70% ethanol (its solvent) had no effect on cell viability. Carboplatin had no effect on OSA cells. Geopropolis exerted a cytotoxic effect on canine osteosarcoma, and its introduction as a possible therapeutic agent *in vivo* could be investigated, providing a new contribution to OSA treatment.

## 1. Introduction

Propolis is a resinous material collected by bees from buds and exudates of the plants and mixed with wax and bee enzymes. It is composed *in natura* of 30% wax bee, 50% resins and vegetable balsams, 10% essential oils, 5% pollen, and 5% other substances [1, 2]. Contrarily, indigenous stingless bees such as *Melipona fasciculata* Smith collect resinous material of plants and add soil or clay, forming the so-called “geopropolis” [3, 4]. 

 Although propolis pharmacological activities have been extensively reported [5], few articles have investigated the geopropolis biological action. The antimicrobial activity of geopropolis produced by *Melipona compressipes fasciculate* was analyzed, and its hydroalcoholic extract decreased to 48.5% the number of colonies of *Streptococcus mutans* in saliva of patients [6]. The anti-inflammatory, antinociceptive, and antitumor activities of geopropolis have also been reported [7, 8]. 

 The activity of geopropolis (extracts and gel) against oral pathogens and on *S. mutans* biofilms was investigated [9]. Geopropolis collected in Palmeirândia, State of Maranhão, Brazil, displayed antimicrobial activities; in addition, a geopropolis-based gel was not toxic in an animal model and showed anti-inflammatory effects. Phenolic compounds, triterpenes, and saponins were found in its chemical composition, which may vary according to the local flora [10].

Several researchers have reported the antitumoral property of propolis both *in vitro *and *in vivo* [11], but little is known concerning geopropolis cytotoxic action towards tumor cells. Osteosarcoma (OSA) or osteogenic sarcoma originated from the central or spinal skeleton is considered the most common and malignant skeletal neoplasm in dogs [12, 13] and is a neoplasm frequently diagnosed in dogs [14]. The behavior of OSA in pet dogs is identical to that of pediatric patients, and it has been considered an excellent model *in vivo* to study human OSA [15–17]. Carboplatin, cisplatin, and doxorubicin are chemotherapeutic agents used to induce remission of the tumor and as a maintenance therapy as well. However, the outcome of chemotherapy in dogs is unpredictable and may not respond to the action of cytotoxic drugs [18, 19].

 Since geopropolis possesses several biological properties, we evaluated a possible antitumor action of geopropolis *in vitro*, using OSA cells and different geopropolis concentrations along different periods of time.

## 2. Materials and Methods

### 2.1. Geopropolis Sample

Geopropolis was produced by *Melipona fasciculata* Smith and was aseptically collected in Palmeirândia, Western Maranhão State, where municipalities are formed by different ecosystems such as mangroves, flooded fields, ponds, forests, and babassu [20].

 Samples were ground, and 30% ethanolic extracts of geopropolis were prepared (80 g of geopropolis, completing the volume to 240 mL with 70% alcohol), in the absence of bright light, moderate shaking, and at room temperature. After 24 hours, extracts were filtered and the dry weight of the extract was calculated (9.3 mg/mL). The extract was placed in an amber bottle and kept refrigerated [10, 21].

### 2.2. Canine OSA Cells and spOS-2 Primary Culture

Dogs were subjected to physical examination, anamnesis, and complete clinical history at the Veterinary Hospital, FMVZ, UNESP, Campus of Botucatu. One dog with cytological OSA diagnosis was selected, and a primary culture of canine OSA cells was used [13]. The dog's owner was informed about the research and procedures and signed a free and enlightened consent Form. This work is in agreement with the Ethical Principles adopted by FMVZ, UNESP, Campus of Botucatu, Brazil (Protocol n. 98/2008).

Tumor fragments were harvested and cells were transferred to 25 cm^2^ flasks containing Dulbecco's Modified Eagle's Medium (DMEM), supplemented with 10% fetal calf serum, penicillin (100 UI/mL), streptomycin (100 *μ*g/mL), and amphotericin-B (2.5 *μ*g/mL). Cells were incubated at 37°C and 5% CO_2_. After confluence, cells were trypsinized and *in vitro* assays were carried out in triplicates. 

 spOS-2 primary cultures were isolated and characterized by biochemical and biomarker panels including alizarin red and by target proteins such as vimentin, cytokeratin, osteocalcin, osteopontin, osterix, and cyclo-oxygenase-2, using flow cytometry. Besides, Cox-2 was also evaluated by immunohistochemistry and divided into Cox-2 positive or Cox-2 negative cultures. Thus, spOS-2 refers to a culture with upregulated Cox-2 expression.

### 2.3. Cytotoxicity Assay

After detachment from the flasks, cells were counted using a haemocytometer and cultivated in a 96-well U-bottomed plate (Corning) at a final concentration of 2 × 10^4^ cells/mL, adding 100 *μ*L/well. 

 Geopropolis was diluted in DMEM medium, and specific dilutions were prepared for each assay in order to achieve 5, 10, 25, 50, and 100 *μ*g. The same procedures were performed with 70% ethanol (geopropolis solvent), in order to obtain 0.03; 0.06; 0.15; 0.29, and 0.59% alcohol, which were the respective concentrations of alcohol in geopropolis concentrations. As a positive control, carboplatin (100 and 200 *μ*Mol·L^−1^) was diluted in DMEM medium [22]. Control cells contained only the medium alone.

Cells were washed twice with PBS and incubated with each stimulus at 37°C and 5% CO_2_ for 6, 24, 48, and 72 h. Cell viability was assessed over time. 

### 2.4. Cell Viability

After each period of time, OSA cell morphology was evaluated microscopically, and cell viability was analyzed by violet crystal method as follows. Cells were incubated with 100 *μ*L violet crystal solution (0.2% diluted in 20% ethanol), which stains live cells. After 10 min, cells were washed four times, and 100 *μ*L of 1% sodium dodecyl sulfate (SDS) was added. The optical density (O.D.) was read in an ELISA reader (Labsystems, Multiskan EX) at 492 nm, and the percentages of cell viability were calculated [23].

### 2.5. Statistical Analysis

Friedman's test was used in order to analyze cell viability in the cultures for each geopropolis concentration according to the time period. Kruskal-Wallis test was used to analyze the time period according to geopropolis concentrations. Statistical significances were accepted when *P* < 0.05, and data represent three similar assays.

## 3. Results

### 3.1. OSA Cells Morphology

**Figure 1:**
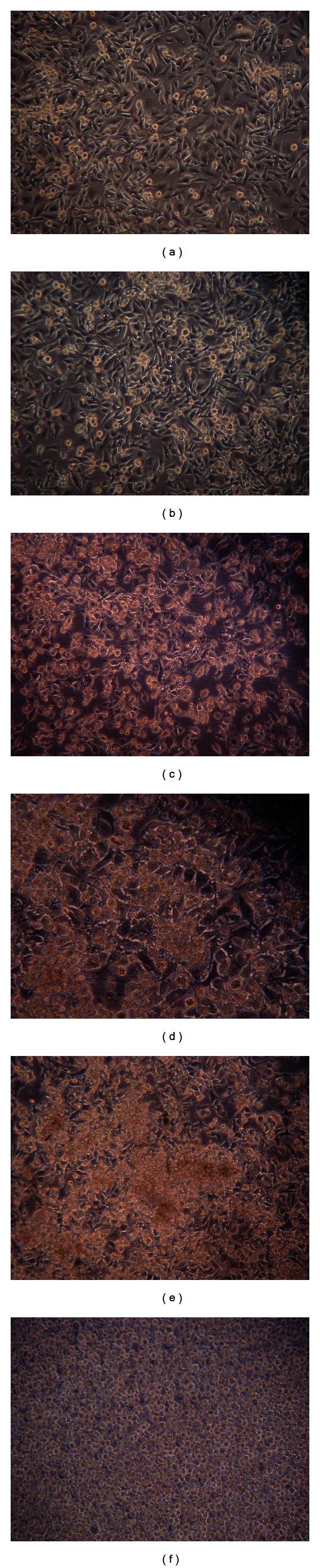
Canine OSA cells (100-fold increase). (a) Control; (b) 70% ethanol; (c) geopropolis 6 h, 50 *μ*g; (d) geopropolis 24 h, 50 *μ*g; (e) geopropolis 48 h, 10 *μ*g; (f) geopropolis 72 h, 10 *μ*g. Data represent three similar assays.

OSA cells are elongated, binucleated, or multinucleated polyhedral or pentagonal cells, showing cytoplasmic granules and vacuoles in most cells [24]. Geopropolis effect on OSA cells may be seen after 6 hours using 50 *μ*g/well (Figure 1(c)); 24 hours and 50 *μ*g (Figure 1(d)); 48 hours and 10 *μ*g (Figure 1(e)), and 72 hours and 10 *μ*g (Figure 1(f)), comparing to control (Figure 1(a)). 70% ethanol in different concentrations had no effect on OSA morphology (Figure 1(b)).

### 3.2. OSA Cells Viability

**Figure 2:**
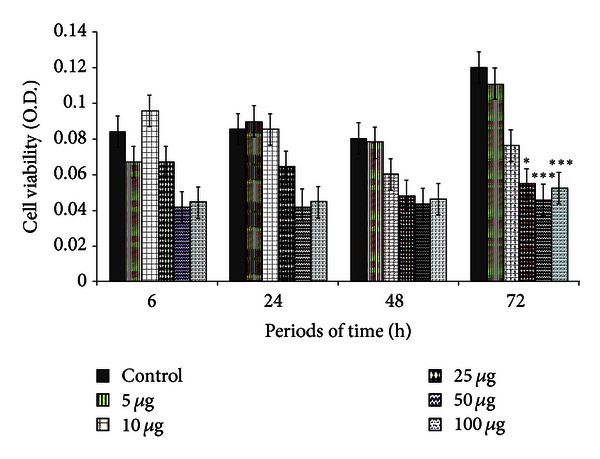
Canine OSA cell viability (optical density) according to geopropolis concentration (5, 10, 25, 50, and 100 *μ*g/well) and periods of time (6, 24, 48, and 72 h). Significantly different from the respective control (*P < 0.05; ***P < 0.0001).

**Figure 3:**
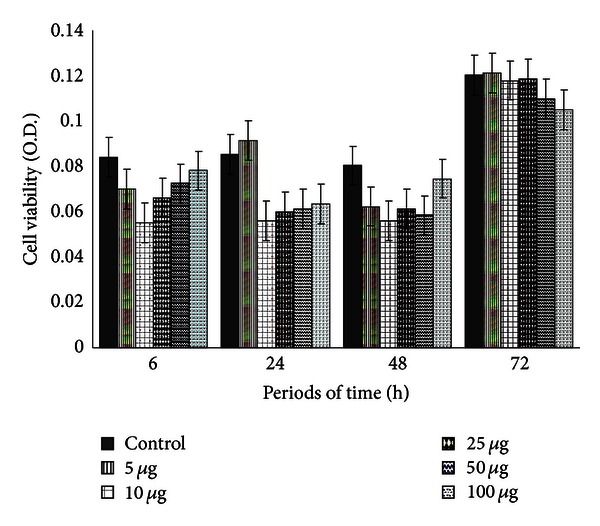
Canine OSA cell viability (optical density) after 6, 24, 48, and 72 h of incubation with different concentrations of 70% ethanol corresponding to geopropolis concentrations (5, 10, 25, 50, and 100 *μ*g/well) (*P* > 0.05).

OSA cells were sensitive to geopropolis in the following periods of time: 6 hours and 50–100 *μ*g/well, 24 h with 50–100 *μ*g, 48 h with 10–100 *μ*g, and 72 h with 10–100 *μ*g, although nonsignificant. A significant decrease in cell viability was seen at 72 h using 25 (*P* < 0.05), 50, and 100 *μ*g/well (*P* < 0.0001) (Figure 2). 70% alcohol showed no effect on cell cultures under study (Figure 3).

### 3.3. Carboplatin Effect on OSA Cells Viability

**Figure 4:**
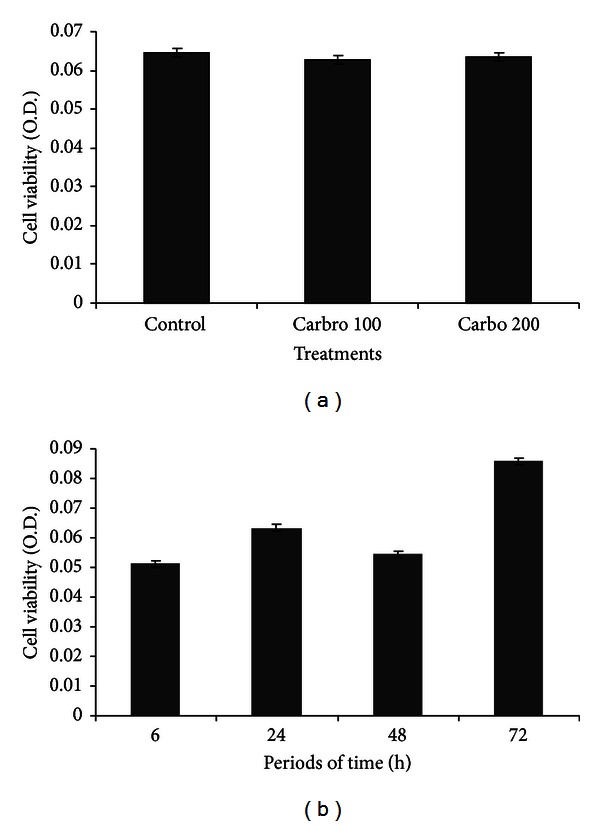
(a) Canine OSA cell viability (optical density) according to carboplatin concentration (carbo—100 and 200 *μ*Mol/L) and different periods of time (b) (*P* > 0.05).

Carboplatin (100 and 200 *μ*Mol·L^−1^) had no effect over the periods of incubation of canine OSA cells with geopropolis (*P* > 0.05) in our assay conditions (Figure 4).

## 4. Discussion

Osteosarcoma is the primary bone tumor most frequently diagnosed in dogs, accounting for more than 80% of cases [14, 25], representing an excellent model *in vivo* to study human OSA, since its biology in dogs is similar to humans [15–17]. This type of cancer accounts for approximately 2% to 5% of all cancers in dogs and less than 1% in humans [26]. 

There have been a great number of researches dealing with propolis and tumor cells both *in vitro* and *in vivo*, using animal or humans models [11], indicating its potential for the development of new antitumor agents [5]. Nevertheless, little is known regarding geopropolis antitumoral activity.

*In vivo* and *in vitro* assays have been performed using hydroalcoholic extract of geopropolis produced by *Melipona fasciculata* on the development of Ehrlich ascitic tumor [7]. *In vitro*, geopropolis decreased the number of tumor cells after incubation with 500 *μ*g/mL for 24 h. In our assays, the cytotoxic action of geopropolis was achieved using 25 *μ*g after a longer period of incubation (72 h). Propolis produced by another stingless bee (*Scaptotrigona* sp.) was efficient against human glioblastoma (U251 and U343) [27]. 

*In vivo*, the pretreatment of mice with geopropolis (50 mg/kg) before the inoculation of tumor cells increased significantly the influx of macrophages into the peritoneal cavity and the release of hydrogen peroxide (H_2_O_2_) and nitric oxide (NO) [7]. Moreover, geopropolis treatment inhibited the number of tumor cells and increased the survival of animals, suggesting that the antitumor effect of geopropolis may be related both to its direct tumoricidal effect and to its ability to recruit macrophages to the tumor focus, with a subsequent production of tumoricidal metabolites.

 The effect of the hydroalcoholic extract of propolis produced by *Scaptotrigona* aff. *postica* was investigated on Ehrlich tumor development in female mice (a single dose 48 h prior to tumor inoculation) [28]. Propolis inhibited tumor development and increased the cell number in the spleen and bone marrow. 

In our work, geopropolis exerted a cytotoxic effect on canine OSA cells, in a dose- and time-dependent manner, and the morphological analysis showed that osteosarcoma cells were sensitive to geopropolis in all periods of time. Its solvent (70% ethanol) had no effect on cell viability, suggesting that the cytotoxic action was exclusively due to geopropolis. The chemical analysis of geopropolis by gas chromatography-mass spectrometry (GC-MS) revealed that the main groups of compounds were pentoses, hexoses, sugar alcohols, uronic acids, disaccharides, alkylresorcinols, and triterpenes, which may be related to the anticancer effects of geopropolis on OSA cells. 

Recently, we verified that propolis produced by Africanized honeybees affected OSA viability after 72 h compared to control, using 50 *μ*g and 100 *μ*g/well, in this same model. A comparison between these data showed that geopropolis was more efficient than propolis since the former had a cytotoxic action at lower concentrations [29]. Previous works of our laboratory reported propolis action on canine venereal transmissible tumor (TVT) [30] and on human laryngeal epidermoid carcinoma (HEp-2) cells [31]. It has been proposed that apoptosis and cell cycle arrest are the main mechanisms by which propolis affects the viability of tumor cells [11]. To date, no evidence of a possible mechanism of action of geopropolis has been proposed.

 Carboplatin has been used as the main chemotherapeutic agent to treat canine OSA; nevertheless, it did not exert a cytotoxic effect in our assays. Three different osteosarcoma cell lines were investigated, showing that these lines were resistant to platinum chemotherapy [32]. Since the lines are monoclonal cell cultures, whose behavior *in vitro* exhibits a reduced aggression in relation to primary cultures [33], one may speculate that OSA culture showed a similar behavior to those resistant to carboplatin.

 There are no works dealing with geopropolis and OSA, and our preliminary data point to a potential role of geopropolis in dogs with osteosarcoma, although further research should be carried out *in vivo* in order to evaluate its therapeutic action.
